# Trophectoderm Biopsy Differentially Influences the Level of Serum β-Human Chorionic Gonadotropin With Different Embryonic Trophectoderm Scores in Early Pregnancy From 7847 Single-Blastocyst Transfer Cycles

**DOI:** 10.3389/fendo.2022.794720

**Published:** 2022-02-18

**Authors:** Yuan Li, Quan Wen, Jingnan Liao, Shujuan Ma, Shuoping Zhang, Yifan Gu, Yi Tang, Keli Luo, Xiaoyi Yang, Guang-Xiu Lu, Ge Lin, Fei Gong

**Affiliations:** ^1^ Institute of Reproductive and Stem Cell Engineering, School of Basic Medical Science, Central South University, Changsha, China; ^2^ Reproductive and Genetic Hospital of CITIC-Xiangya, Changsha, China; ^3^ NHC Key Laboratory of Human Stem Cell and Reproductive Engineering, Central South University, Changsha, China

**Keywords:** β-hCG, trophectoderm biopsy, trophectoderm score, preimplantation genetic test, obstetric and neonatal outcomes

## Abstract

**Objective:**

To evaluate whether trophectoderm (TE) biopsy differentially influence the level of serum β-human chorionic gonadotropin (β-hCG) with different TE-scored blastocysts transferred in early pregnancy.

**Methods:**

This retrospective cohort study contained 7847 single-blastocyst transfer cycles executed between January 2019 and June 2020, including 2657 preimplantation genetic testing (PGT) cycles and 5190 *in vitro* fertilization (IVF) or intracytoplasmic sperm injection (ICSI) cycles. All cycles were classified into biopsy and control groups, and further stratified based on the TE morphological scores into three subgroups: grades A, B, and C for TE scores, respectively. Intra-group and inter-group analyses were performed on serum β-hCG levels on the 12th day after blastocyst transfer (HCG_12_), and obstetric and neonatal outcomes.

**Results:**

For cycles with a live birth, in grade A TE score subgroups, the HCG_12_ level did not exhibit statistical significance between the control and biopsy groups after adjustment (769 mIU/mL vs. 753 mIU/mL, P=0.631). In contrast, in grade B and C TE score subgroups, the control group showed a significantly higher level of HCG_12_ relative to the biopsy group (690 mIU/mL vs. 649 mIU/mL, P=0.001; 586 mIU/mL vs. 509 mIU/mL, P<0.001, respectively). We observed no statistically significant differences in obvious adverse obstetric and neonatal outcomes between the same TE-score subgroups of the biopsy group and control group.

**Conclusions:**

While blastocysts with higher TE grades produced higher serum β-hCG levels in early pregnancy, TE biopsy might exert a negative impact on serum β-hCG levels by blastocysts with a grade-B TE score and below. TE biopsy did not increase the risk for adverse obstetric and neonatal outcomes.

## Introduction

Preimplantation genetic testing (PGT) is an assisted reproductive technology (ART) procedure that is used to investigate the genetic differences in embryos produced *in vitro* fertilization (IVF) or IVF in combination with intracytoplasmic sperm injection (ICSI) (1), and is carried out for selecting genetically normal transferable embryos so as to improve live-birth rates (LBRs) with IVF (2, 3). TE biopsy involves removing several TE cells at the blastocyst stage; this technique has undergone a long history of development (4). TE biopsy, an invasive manipulation, is increasingly used in clinical practice, but the assessment of potential long-term safety concerns on humans is currently inadequate, it is thus imperative and advisable to evaluate the potential impact of this modality (5).

Human chorionic gonadotropin (hCG), a heterodimeric glycoprotein composed of alpha and beta subunits (6), is the most important early embryo-derived signal and plays a pivotal role in implantation (7). It exerts autocrine regulation of trophoblast invasion (8–10) as well as paracrine control of the endometrial environment to support embryo attachment and implantation (11). Serum β-hCG can be detected in maternal blood about one week after fertilization and is often regarded as a signal of maternal recognition of pregnancy (7, 12); it is also applied to prospectively distinguish viable pregnancies from ectopic pregnancies and spontaneous abortions (13).

Serum β-hCG is produced primarily by differentiated syncytiotrophoblasts of trophectoderm (TE) (14) and represents TE function (15). Whether biopsy would affect serum β-hCG levels remains controversial. In 1991, researchers found decreased hCG secretion in biopsied embryos when more than 10 TE cells were removed (16); and a recent study showed that TE biopsy reduced the level of maternal peripheral blood serum β-hCG in early pregnancy (17). In contradistinction, subsequent retrospective studies drew the conclusion that PGT appeared not to affect the levels of serum β-hCG (15, 18).

TE scores, based on Gardner’s morphological scoring system (19), can reflect junctional tightness of TE cells. It is natural to conjecture that blastocysts with high TE scores (representing a larger number of total TE cells and of higher quality), would be less affected by biopsy, and it has been reported that the reproductive potential of blastocysts with poor TE quality were more likely to be affected by biopsy (20). Previous investigators have not considered differences in TE quality when evaluating its impact on serum β-hCG (15, 17, 18), and this might account for the inconsistency in conclusions. We therefore allocated embryos following their TE scores so as to explore whether serum β-hCG levels were affected by TE biopsy to different degrees in early pregnancy.

In addition, we appreciate that trophoblast cells continue to develop and then form the placenta, and that abnormal trophoblast may contribute to adverse obstetrical outcomes such as preeclampsia (21). In the present study, we investigated whether TE biopsy differentially influenced the levels of serum β-hCG produced by embryos with different embryonic TE scores in early pregnancy, and then assessed whether biopsy would affect certain obstetrical and neonatal outcomes.

## Materials and Methods

### Study Design and Patients

This study was a retrospective cohort study. A total of **12,950** frozen single-blastocyst transfer (SBT) cycles were performed from January 2019 to June 2020 in the Reproductive and Genetic Hospital of CITIC-Xiangya. According to the patients whether receiving PGT, these cycles were divided into two groups: the biopsy group (PGT) and the control group (IVF/ICSI). Indications of PGT were as follows: advanced maternal age (age ≥35 years), chromosomal abnormality, monogenic disease, recurrent spontaneous abortion (RSA), or recurrent implantation failure (RIF). According to inclusion and exclusion criteria, 2657 cycles in the biopsy group and 5190 cycles in the control group were ultimately included in the analysis. The inclusion criteria were as follows: (i) having a positive serum β-hCG result detected on day 12 after single- embryo transfer (ET); and (ii) that the data on transferred embryos and pregnancy outcome were available. The exclusion criteria included (i) exogenous hCG applied to luteal support after transplantation and (ii) cycles with multiple pregnancies**;** (iii) transferred blastocyst with a grade C inner cell mass (ICM) score. More details are presented in **Supplementary Figure 1**. We obtained ethical approval for this study from the ethics committee of CITIC-Xiangya, People’s Republic of China (LL-SC-2021-015).

### Clinical Procedures

Ovarian-stimulation protocols were performed as described by Tan et al. (22). Ovulation was triggered applying 5,000–10,000 IU hCG (Pregnyl Merck) when two-thirds of the follicles reached 18 mm. Transvaginal ultrasound-guided oocyte retrieval took place 34–36 hours later. Oocytes were fertilized by IVF or ICSI 4–6 hours after oocyte retrieval, and ICSI was performed in all PGT cycles. Normal fertilization was confirmed by the presence of two pronuclei and two polar bodies at 16–18 hours after insemination or injection. Embryos were cultured in G1.5/G2.5 sequential media (Vitrolife) to blastocyst stage in a COOK mini-incubator at 37°C, with a humidified atmosphere of 6% CO_2_, 5% O_2_, and 89% N_2_ in air.

On the morning of day 5 or day 6 after insemination, blastocyst morphology was assessed according to Gardner’s scoring system, and the blastocyst ICM and TE were graded (A-C) as follows. Subtle adjustments to the scoring criteria, TE with many cells forming a cohesive epithelium was assigned an A grade, TE with few cells forming a loose epithelium assigned a B grade, and TE with very few large cells assigned a C grade; ICM with many cells tightly packed was assigned an A grade, ICM with several cells loosely grouped assigned a B grade, and ICM with very few cells assigned a C grade (23). Blastocysts with a grade ≥4BB were considered to be high-quality blastocysts. Blastocyst biopsy was performed on the morning of day 6. The blastocyst was immobilized using the holding pipette, the herniating TE was drawn into a biopsy pipette (internal diameter, 30 mm), and a Zilos TK laser (Hamilton Thorne) separated a piece of TE (about 5–10 cells) away from the ICM. Blastocysts were vitrified within 1–2 hours after biopsy and thawed as described in our previous study (24). Vitrification and thawing of blastocysts in IVF/ICSI cycles was performed using the same method.

For the frozen-embryo transfer cycle, only one embryo was transferred to each patient. Embryos were warmed using a commercially available warming solution (Kitazato Biopharma). After warming, the embryos were transferred to G1.5/G2.5 medium and cultured for 2–6 h. Only the blastocysts that re-expanded after warming were considered as suitable for transfer. The blastocysts were transferred 5 days after ovulation in a natural cycle or 5 days after progesterone supplementation in a hormone replacement treatment cycle. Luteal support was applied when the dominant follicle disappeared in a natural cycle or satisfactory endometrial development (thickness≥ 8 mm, confirmed by ultrasonographic examination) in a hormone replacement treatment cycle.

### Serum β-hCG Measurement and Outcome Definitions

Serum β-hCG levels were detected on the 12th day after ET, and serum β-hCG≥7 mIU/ml was regarded as positive. Clinical pregnancy was defined as the presence of an intrauterine gestational sac on the day 28 after ET. Biochemical pregnancy referred to a positive serum β-hCG level but no gestational sac detected *via* ultrasound. Miscarriage was defined as a pregnancy loss prior to 20 weeks of gestation, and stillbirth as the death of a fetus prior to the complete expulsion or extraction from its mother after 20 completed weeks of gestational age. Ectopic pregnancy was defined when the gestational sac appeared outside the uterine cavity. A live birth was defined as an infant born with signs of life. Oligohydramnios was defined as amniotic fluid index (AFI) ≤5 cm and polyhydramnios was defined as AFI > 24 cm. Low birth weight (LBW) was defined as birth weight under 2,500 g, very low birth weight (VLBW) was defined as under 1,500 g, and macrosomia was defined as over 4,000 g.

### Statistical Analysis

All statistical analyses were executed using statistical software SPSS 26.0 (IBM), and P < 0.05 was considered significant. Continuous data were presented as mean and standard deviation or median (interquartile range). Mann–Whitney U-test and Kruskal–Wallis test were applied to assess intergroup differences. Categorical data were presented as percentages (counts) and analyzed by the Chi-squared test, continuity-corrected Chi-squared test, or Fisher’s exact test. A generalized linear model and logistic regression were performed to assess the impact of potential variables on target parameters, where applicable. Receiver operating characteristics (ROC) curve analysis was used to evaluate the performance of serum β-hCG in predicting a live birth. The optimal cutoff value was calculated using Youden’s index with the highest sum of sensitivity and specificity.

## Results

### Demographics and Baseline Values

The demographics and baseline values of the biopsy and control groups are compared in **Table 1**. The body mass index (BMI) and endometrial thickness on the day before transfer did not exhibit significant differences between the two groups. Significant differences were, however, observed in maternal age, basal hormone levels (i.e., follicle-stimulating hormone, luteinizing hormone, and estradiol), and endometrial preparation protocols. As expected, advanced age and the prevalences of chromosomal abnormality, monogenic disease, RSA, and RIF were significantly higher in the biopsy group (with all p-values <0.001). The biopsy group also exhibited a significantly higher rate for the transfer of high-quality embryos relative to the control group (80.05% vs. 61.29%, P<0.001), and the median serum β-hCG value on the day 12 after blastocyst transfer (HCG_12_) in the biopsy group was significantly higher when compared to the control group (495 mIU/mL vs. 455 mIU/mL, P<0.001).

**Table 1:** Baseline characteristics of cycles with HCG_12_ ≥7 mIU/mL.

Characteristic	Biopsy group	Control group	p-value
FET cycles, n	2657	5190	
Age (years)	33 (30-37)	32 (29-35)	<0.001
BMI (kg/m^2^)	21.63 (20.03-23.24)	21.78 (20.03-23.43)	0.131
Duration of infertility (years)	2 (1-4)	3 (2-5)	<0.001
Basal values			
FSH (mIU/mL)	5.68 (4.75-6.75)	5.49 (4.54-6.53)	<0.001
LH (mIU/mL)	3.46 (2.51-4.67)	3.32 (2.33-4.71)	0.001
E_2_ (pg/mL)	34 (26-44)	33 (24-44)	0.002
Endometrial thickness on the day before transfer (mm)	11.80 (10.50-13.20)	11.80 (10.50-13.10)	0.474
Advanced age, % (n)	41.21 (1095)	26.82 (1392)	<0.001
Chromosomal abnormality, % (n)	30.30 (805)	0.96 (50)	<0.001
Monogenic disease, % (n)	15.47 (411)	3.93 (204)	<0.001
RSA, % (n)	27.63 (734)	3.37 (175)	<0.001
RIF, % (n)	4.67 (124)	2.43 (126)	<0.001
Rate of high-quality embryos transferred, % (n)	80.05 (2127)	61.29 (3181)	<0.001
Endometrial preparation protocols, % (n)			<0.001
NC-FET	85.70 (2277)	78.03 (4050)	
HT-FET	7.30 (194)	11.70 (607)	
Down-regulating HT-FET	7.00 (186)	10.27 (533)	
HCG_12_ (mIU/mL)	495 (215-760)	455 (130-735)	<0.001

Variables are expressed as medians (interquartile range) unless otherwise stated. FET, frozen embryo transfer; BMI, body mass index; FSH, follicle-stimulating hormone; LH, luteinizing hormone; E_2,_ estradiol; RSA, recurrent spontaneous abortion; RIF, recurrent implantation failure NC, natural cycle; HT, hormone therapy; HCG_12_, serum β human chorionic gonadotropin on 12 days after blastocyst transfer.

### ROC Curve Analysis of HCG_12_ in Predicting a Live Birth in the Biopsy and Control Groups

We performed a ROC curve analysis to assess HCG_12_ levels in predicting a live birth. In the biopsy group, the area under the ROC curve was 0.886 (95% CI, 0.870–0.902), while the optimal cutoff value was 266 mIU/mL (with a sensitivity of 90.9% and specificity of 75.0%). In the control group, the area under the ROC curve was 0.874 (95% CI, 0.864–0.885), and the optimal cutoff value was 299 mIU/mL (with a sensitivity of 89.1% and specificity of 75.0%) (**Supplementary Figure 2**). Serum β-hCG 12 days after ET revealed a high diagnostic accuracy in both groups, with the optimal cutoff value higher in the control group.

### Baseline Characteristics of Cycles With Live Births

To better evaluate the impact of embryonic biopsy on maternal serum β-hCG levels, and to eliminate possible counteracting effects of biochemical and ectopic pregnancies and miscarriages, only cycles with live births were used in the comparisons. A total of 4907 cycles were included in the analysis: 1873 cycles from the biopsy group and 3034 cycles from the control group, and there were no duplicate patients. BMI and endometrial thickness on the day before transfer showed no differences between groups, while advanced age and the prevalences of chromosomal abnormality, monogenic disease, RSA, and RIF were identified as significantly different. Detailed comparisons were shown in (**Table 2**). However, the median HCG_12_ value was no longer significantly different between the biopsy group and control group (616 mIU/mL vs. 634 mIU/mL, P=0.136), and the HCG_12_ levels were even reversed. Intriguingly, the biopsy group exhibited a significantly higher rate of high-quality embryos transferred compared to the control group (83.72% vs. 67.24%, P<0.001).

**Table 2:** Baseline characteristics of cycles resulting in live births.

Characteristic	Biopsy group	Control group	p-value
FET cycles, n	1873	3034	
Age (years)	33 (29-37)	31 (29-34)	<0.001
BMI (kg/m^2^)	21.64 (20.03-23.33)	21.78 (20.03-23.44)	0.447
Duration of infertility (years)	2 (1-4)	3 (2-5)	<0.001
Basal values			
FSH (mIU/mL)	5.67 (4.73-6.75)	5.42 (4.47-6.44)	<0.001
LH (mIU/mL)	3.50 (2.52-4.69)	3.37 (2.33-4.79)	0.028
E_2_ (pg/mL)	34.00 (26.00-44.61)	32.00 (24.00-44.00)	<0.001
Endometrial thickness on the day before transfer (mm)	11.90 (10.60-13.20)	11.90 (10.70-13.10)	0.824
Advanced age, % (n)	39.19 (734)	21.16 (642)	<0.001
Chromosomal abnormality, % (n)	30.86 (578)	0.99 (30)	<0.001
Monogenic disease, % (n)	15.59 (292)	3.89 (118)	<0.001
RSA, % (n)	27.66 (518)	2.60 (79)	<0.001
RIF, % (n)	4.06 (76)	2.27 (69)	<0.001
Rate of high-quality embryos transferred, % (n)	83.72 (1568)	67.24 (2040)	<0.001
Endometrial preparation protocols, % (n)			<0.001
NC-FET	87.77 (1644)	78.21 (2373)	
HT-FET	6.73 (126)	11.96 (363)	
Down-regulating HT-FET	5.50 (103)	9.82 (298)	
HCG_12_ (mIU/mL)	616 (427-847)	634 (441-851)	0.136

Variables are expressed as medians (interquartile range) unless otherwise stated. FET, frozen embryo transfer; BMI, body mass index; FSH, follicle-stimulating hormone; LH, luteinizing hormone; E_2,_ estradiol; RSA, recurrent spontaneous abortion; RIF, recurrent implantation failure NC, natural cycle; HT, hormone therapy; HCG_12,_ serum β human chorionic gonadotropin on 12 days after blastocyst transfer.

### HCG_12_ Levels Among Different TE-Score Subgroups With Live Births

The proportion of high-quality embryos was significantly different between the biopsy and control groups, and this may bias the data analysis on serum β-hCG levels. Therefore, we stratified the two groups based on the TE morphological scores in three subgroups: the grade A, B, and C TE score, respectively. Detailed demographics and baseline data are shown in **Supplementary Table 1**. The median HCG_12_ values in grade A, B and C TE score subgroup were 761 mIU/mL, 624 mIU/mL, and 483 mIU/mL in the biopsy group and 725 mIU/mL, 663 mIU/mL, and 565 mIU/mL in the control group, both P values were less than 0.001 among multiple subgroups.

**Figure 1:**
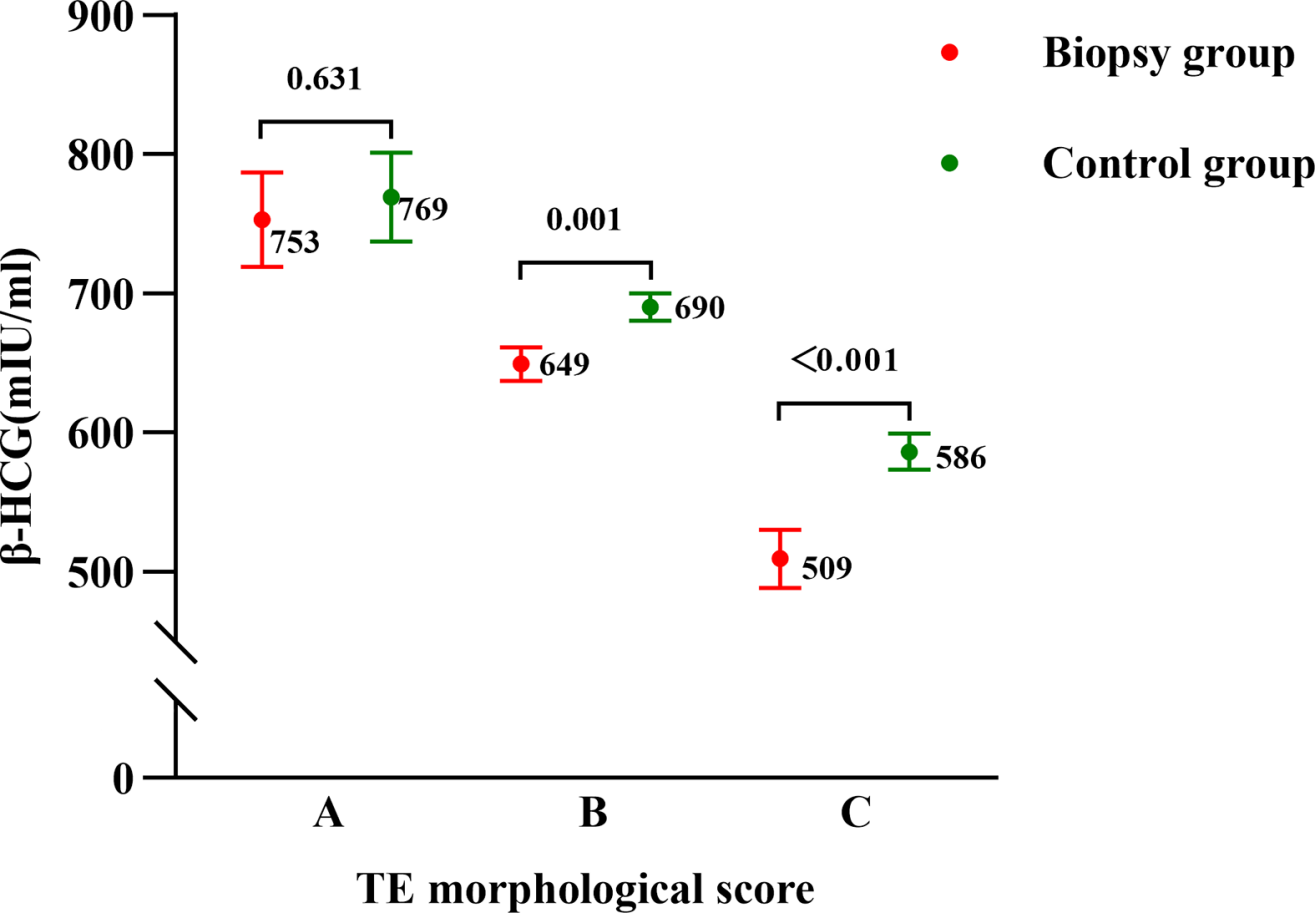
Serum β-HCG levels on the 12th day after blastocyst transfer in the biopsy and control groups as stratified by morphological TE scores. Points and errors bars represent marginal means and standard errors, respectively.

As for same grade of TE score subgroup comparisons between the biopsy group and control group, we used generalized linear models to control for confounders and calculated adjusted means (marginal means). Potential influencing factors that included maternal age, BMI, duration of infertility, basal hormone levels, endometrial thickness on the day before transfer and endometrial preparation protocols were included in the models. After adjustment, in grade A TE score subgroups, the average HCG_12_ level did not show a statistical difference between the control group and biopsy group (769 mIU/mL vs. 753 mIU/mL, P=0.631); while in grade B and C TE score subgroups, the control group exhibited a significantly higher level of HCG_12_ than the biopsy group (690 mIU/mL vs. 649 mIU/mL, P=0.001; 586 mIU/mL vs. 509 mIU/mL, P<0.001, respectively (**Figure 1**).

### HCG_12_ for Predicting a Live Birth With Different Grades of TE Score Blastocyst Transfer

**Figure 2:**
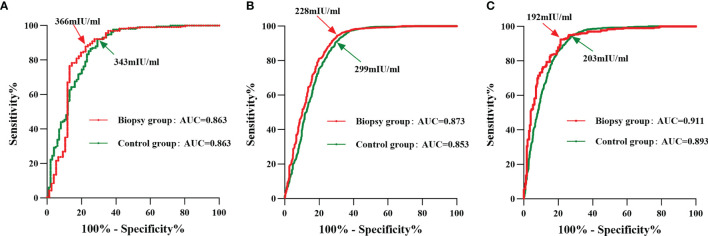
ROC curve analysis of HCG_12_ in predicting a live birth as a function of different grades of TE-scored blastocyst transfers. Red line, biopsy group; green line, control group. The points indicated by arrows represent the optimal cutoff values with the highest sum of sensitivity and specificity. **(A)**, grade-A TE score of transferred blastocysts; **(B)**, grade-B TE score of transferred blastocysts; **(C)**, grade-C TE score of transferred blastocysts.

In order to explore the HCG_12_ with regard to the predictive value for live births with different grades of TE-scored blastocysts transferred in both the biopsy and control group, ROC curve analysis was performed. The optimal cutoff value of HCG_12_ in the biopsy subgroups with grades A, B, and C TE scores were 366 mIU/mL, 228 mIU/mL, and 192 mIU/m, respectively. The control group had a lower optimal cutoff value (343 mIU/m) in the grade A TE score subgroup compared with the same subgroup in biopsy group. However, in the grade B and C TE score subgroups, the optimal cutoff values were higher in the control group at 299 mIU/mL and 203 mIU/mL, respectively (**Figure 2** and **Supplementary Table 2**).

### Obstetric and Neonatal Outcomes

A small fraction of live-birth cycles with in complete data of obstetric and neonatal outcomes was further excluded in this part of the analysis, finally resulting in 1853 cycles in the biopsy group and 3006 cycles in the control group. For each outcome, we performed generalized linear regression or logistic regression analysis to explore the impact of TE biopsy on it. After adjusting for confounders that included maternal age, BMI, duration of infertility, basal hormone levels, endometrial thickness on the day before transfer and endometrial preparation protocols, maternal outcomes including hypertensive disorders of pregnancy (HDP), edema, gestational diabetes mellitus (GDM), anemia, placenta previa, placental abruption, abnormal amniotic fluid (i.e., oligohydramnios and polyhydramnios), and fetal distress and other pregnancy complications and neonatal outcomes that included congenital malformation, LBW, VLBW, macrosomia, and birth length were not statistically different between the same TE-score subgroups of the biopsy group and control group (**Table 3**). However, in the biopsy group, birth weight was significantly higher (adjusted odds ratio (aOR) 1.12, 95% CI 1.01-1.24; P= 0.037) when transferring grade A TE-score blastocysts and the probability of cesarean section was significantly lower (aOR 0.81, 95% CI 0.68-0.96; P=0.015) when transferring grade B TE-score blastocysts. On the other hand, we only noted in intragroup comparisons among multiple TE-score subgroups of the biopsy and control groups that the higher TE grade subgroup manifested a statistical tendency for greater birth weight in the biopsy group (P=0.004).

**Table 3:** The obstetric and neonatal outcomes of live births.

Characteristic	TE morphological score												p.value_(ABC)_	
	A				B				C					
	Biopsy group	Control group	OR(95CI)	p.value	Biopsy group	Control group	OR(95CI)	p.value	Biopsy group	Control group	OR(95CI)	p.value	Biopsy group	Control group
Live births, n	230	212			1322	1816			301	978				
HDP, (n)	5.65(13)	7.08(15)	0.73(0.31-1.74)	0.482	3.48(46)	4.46(81)	0.83(0.56-1.23)	0.352	4.98(15)	4.19(41)	1.46(0.76-2.81)	0.258	0.189	0.180
Edema, (n)	9.57(22)	8.49(18)	1.32(0.65-2.67)	0.444	10.59(140)	9.97(181)	1.06(0.83-1.36)	0.630	11.96(36)	9.30(91)	1.48(0.96-2.29)	0.079	0.661	0.715
GDM, (n)	23.04(53)	24.06(51)	0.85(0.52-1.40)	0.527	22.24(294)	19.55(355)	1.07(0.89-1.29)	0.452	23.26(70)	21.17(207)	1.01(0.73-1.41)	0.949	0.910	0.233
Anemia, (n)	6.09(14)	8.49(18)	0.66(0.30-1.47)	0.312	4.77(63)	4.85(88)	1.03(0.73-1.46)	0.855	5.65(17)	4.91(48)	1.24(0.68-2.23)	0.484	0.620	0.070
Placenta previa, (n)	0.43(1)	0.00(0)	–	–	0.53(7)	0.55(10)	0.64(0.22-1.83)	0.403	0.33(1)	0.41(4)	0.77(0.06-9.37)	0.834	1.000	0.754
Placental abruption, (n)	0.00(0)	0.00(0)	–	–	0.00(0)	0.06(1)	–	–	0.32(1)	0.10(1)	–	–	0.287	1.000
Amniotic fluid, (n)													0.175	0.588
Normal			reference				reference				reference			
Oligohydramnios	0.43(1)	1.42(3)	1.65(0.08-33.71)	0.746	1.13(15)	1.21(22)	0.93(0.46-1.88)	0.844	2.66(8)	1.23(12)	2.29(0.87-6.02)	0.093		
Polyhydramnios	0.00(0)	0.00(0)	–	–	0.30(4)	0.33(6)	0.75(0.19-2.97)	0.683	0.33(1)	0.72(7)	0.64(0.08-5.44)	0.686		
Fetal distress, (n)	0.00(0)	0.00(0)	–	–	0.00(0)	0.00(0)	–	–	0.00(0)	0.31(3)	–	–	–	0.242
Other pregnancy complications, (n)	1.74(4)	1.89(4)	1.07(0.23-5.03)	0.928	1.97(26)	1.87(34)	1.22(0.7-2.14)	0.482	1.00(3)	1.33(13)	0.52(0.13-2.07)	0.355	0.590	0.537
Congenital malformation, (n)	2.17(5)	4.25(9)	0.71(0.19-2.58)	0.601	2.34(31)	1.76(32)	1.46(0.86-2.48)	0.165	2.99(9)	1.84(18)	1.52(0.64-3.61)	0.344	0.778	0.071
Mode of delivery, (n)													0.794	0.966
Cesarean	69.57(160)	72.17(153)	0.87(0.55-1.37)	0.536	70.42(931)	73.02(1326)	0.81(0.68-0.96)	0.015	72.09(217)	72.90(713)	0.98(0.72-1.34)	0.916		
Vaginal	30.43(70)	27.83(59)			29.58(391)	26.98(490)			27.91(84)	27.10(265)				
Birth weight (kg)	3.42 ± 0.54	3.30 ± 0.52	1.12(1.01-1.24)	0.037	3.32 ± 0.50	3.35 ± 0.51	0.98(0.94-1.02)	0.235	3.29 ± 0.50	3.34 ± 0.50	0.95(0.89-1.02)	0.156	0.004	0.638
VLBW, (n)	1.30(3)	0.94(2)	1.61(0.23-11.13)	0.630	0.61(8)	0.66(12)	0.70(0.26-1.85)	0.470	0.66(2)	0.92(9)	0.57(0.11-3.11)	0.517	0.488	0.657
LBW, (n)	3.48(8)	7.08(15)	0.49(0.19-1.27)	0.143	3.63(48)	3.80(69)	0.95(0.64-1.41)	0.790	4.32(13)	3.58(35)	1.11(0.55-2.22)	0.771	0.832	0.052
Macrosomia, (n)	8.26(19)	3.77(8)	1.77(0.69-4.5)	0.234	6.20(82)	6.55(119)	0.96(0.7-1.31)	0.790	5.32(16)	5.83(57)	1.02(0.56-1.85)	0.958	0.366	0.251
Birth length (cm)	50.29 ± 1.46	50.04 ± 1.34	1.18(0.82-1.71)	0.374	50.11 ± 1.24	50.08 ± 1.20	1.06(0.95-1.18)	0.333	50.07 ± 1.36	50.05 ± 1.32	1.06(0.86-1.32)	0.568	0.287	0.898

Variables are expressed as mean ± standard deviation unless otherwise stated. CI, confidence interval; HDP, hypertensive disorders of pregnancy; GDM, gestational diabetes mellitus; LBW, Low birth weight; VLBW, very low birth weight. p.value_(ABC)_ indicated a comparison among different TE-score subgroups of biopsy or control groups.

## Discussion

PGT is becoming more widely used in recent years. More studies have focused on the safety of use of embryo biopsy and its impact on obstetric and neonatal outcomes, and regarded serum β-hCG as a good evaluation indicator of influence resulting from biopsy. Lu et al. (17) found that TE biopsy reduced the level of serum β-hCG in early pregnancy, although the baseline characteristics in their study were significantly different between biopsy and control groups. Wu et al. (15) used propensity score matching to balance the baseline and drew the conclusion that TE biopsy did not affect serum β-hCG levels. The confounding factors they considered included parental ages, BMI, AMH levels, duration of infertility, endometrial thickness five days before transfer, number of previous gestations and transfers, and days of ET. However, further appraisal needs to be provided as to whether these factors actually affected serum β-hCG levels in early pregnancy, or whether other factors also need to be considered (such as TE scores). Many studies have revealed blastocyst morphology to be linked with serum β-hCG levels (25–27), but the impact of TE grade on individual β-hCG level is not clear. In this study, for cycles resulting in a live birth, we found that HCG_12_ levels were not significant different between the biopsy group and control group, while the rate of high-quality embryos transferred was quite different—and this may affect the result. Therefore, we first explored the effect of TE quality on the level of serum β-hCG levels in early pregnancy.

As expected, HCG_12_ levels showed a statistically significant difference among different TE score subgroups in both biopsy and control groups: grade A was highest, next was grade B, grade C was lowest between them. Correspondingly, the optimal cutoff value of HCG_12_ predicting a live birth in above two groups, was decreased in the following order: grade A subgroup > grade B subgroup > grade C subgroup. This result suggested that blastocysts with advanced TE quality possessed a stronger β-hCG secretory capacity, and that what may be needed is setting a higher cutoff value for predicting a live birth in cycles with transfers of high-grade TE-scored blastocysts. Embryo actively secrete hCG to promote the process of implantation after entering into the uterine cavity (28). As the invasion signal of cytotrophoblast cells, hCG is related to the states characterized by cytotrophoblast cells or invasion (29). We speculated that the production of β-hCG in early pregnancy would feedback the amount of TE and the ability to invade the endometrium of blastocysts. Higher-quality TE blastocysts have more TE cells so that the amount of β-hCG produced during the early pregnancy was relatively higher. Similarly, higher-quality TE blastocysts have a stronger ability to invade the endometrium, requiring a higher level of β-hCG to reach a live birth in order to show its TE invasion ability.

Although previous data at our center showed that the implantation rate decreased with a greater number of biopsied TE cells for blastocysts with grade B and grade C TE scores (20), the rate did not decrease even when 16–41 TE cells were removed from the blastocysts with a grade A TE score. This suggested that TE biopsy approach may impair reproductive potential of the embryo with grade B or grade C TE scores. This conclusion is well supported by our present study, as the biopsy group had a statistically significantly lower level of HCG_12_ than the control group when grade B and C TE-score blastocysts were transferred, while no significant differences were found in the grade A TE-score subgroup. And a lower cutoff value for serum β-hCG in predicting a live birth with PGT appeared only available for blastocysts with poor TE quality (i.e., grade B and C) rather than all embryos. Some investigators previously attempted to explore the impact of TE biopsy on serum β-hCG levels, but reached discordant conclusions; with one of the possible reasons being that subgroup analyses by categorizing the grades of TE were not performed (15, 17, 18). On the basis of a large sample size, we undertook subgroup analyses by blastocyst TE grade and drew a more nuanced and reliable conclusion. The TE biopsy impaired the β-hCG secretory capacity of blastocysts with poor TE quality, while such an adverse impact was not present with good TE quality (i.e., grade A) blastocysts. Thus, although non-invasive PGT for prioritizing embryos has gradually become a reality, there is still a long way to go with respect to its clinical applications (30). Before that happens, we should exercise caution with embryo biopsy, especially with manipulation of blastocysts with poor TE quality.

Blastocyst trophoblast cells are destined to form the placenta, as TE quality and biopsy performance exert an influence on serum β-hCG secretion; and these may adversely affect placenta-related and other pregnancy outcomes. It has been reported that TE quality significantly affected ongoing pregnancy and miscarriage rates (31, 32), but was not observed to increase the risks of adverse obstetric and perinatal outcomes (33, 34). A recent study indicated that birthweight would diminish with decreasing TE quality, and that embryos with TE Grade A had a higher risk of a large-for-gestational-age baby (35). We obtained similar results showing that TE morphological score was significantly related to birth weight in the biopsy group but did not affect other obstetric and neonatal outcomes. The impact of biopsy on obstetric and perinatal outcomes is likewise unknown. Makhijani et al. (36) found that the odds of HDP increased in FET cycles with the transfer of TE-biopsied blastocysts. Zhang et al. (37) compared obstetric and neonatal outcomes of 357 cycles that resulted in live birth and found an augmented risk of preeclampsia associated with TE biopsy. Our center previously conducted a follow-up of 1,721 children, and except for a significantly higher cesarean section rate in the PGT group relative to the control group for twins, no significant differences were noted in other neonatal outcomes between the two groups (24). Two large studies have recently obtained similar results that biopsy did not increase the risk of adverse obstetric and neonatal outcomes with frozen-thawed SBT (17, 38). In this study, we controlled for potential confounders, and only found TE biopsy was associated with higher birthweight (when transferring grade A TE-score blastocysts) and increased rate of cesarean section (when transferring grade B TE-score blastocysts), but the actual difference was quite small and may not be clinically significant. No significant differences were observed in other outcomes between the biopsy and control subgroups with same grade. To the best of our knowledge, we have with this study presented the largest number of frozen SBT cycles with deliveries so as to assess the safety of contemporary PGT practice. Our data further supported the notion that TE biopsy and low TE morphological score did not increase the risk of adverse obstetric and neonatal outcomes.

Some limitations existed with respect to our study. First, this was a retrospective study and was prone to baseline bias. Furthermore, current PGT and routine IVF/ICSI were targeted at different populations with fertility requirements. Thus, we performed a generalized linear model and logistic regression to reduce the bias from baseline characteristics. Nevertheless, the inhomogeneity of the groups was still the limitation of this study. On the other hand, the influences of some basic clinical diseases on obstetric and neonatal outcomes cannot be fully dismissed; thus, the results still need to be treated with caution. In the future, we will employ a prospective study design to exclude disturbances from baseline characteristics and basic diseases, and this should help to corroborate this study’s validity regarding the impact of TE biopsy on obstetric and neonatal outcomes.

In summary, we observed that TE morphological score was related to serum β-hCG levels in early pregnancy and that blastocysts with higher TE grades produced more serum β-hCG. We also found that TE biopsy had a negative impact on serum β-hCG levels by blastocysts with a grade B TE score and below. However, for embryos with good or poor TE quality, TE biopsy did not increase the risk of adverse obstetric or neonatal outcomes.

## Data Availability Statement

The original contributions presented in the study are included in the article/**Supplementary Material**. Further inquiries can be directed to the corresponding author.

## Ethics Statement

The studies involving human participants were reviewed and approved by ethics committee of CITIC-Xiangya, People’s Republic of China (LL-SC-2021-015). Written informed consent for participation was not required for this study in accordance with the national legislation and the institutional requirements.

## Author Contributions

YL and QW conceived and coordinated the study, analyzed the data and drafted the article. JL and SM analyzed and verified the data. SZ, YG, YT, KL, XY revised the article. G-XL and GL designed and guided the study. FG designed and guided the study, and revised the article. All authors reviewed the results and approved the final version of the manuscript.

## Funding

This study was funded by the National Key Research & Developmental Program of China (2018YFC1004901) and the Natural Science Foundation of Hunan Province (2018JJ6088).

## Conflict of Interest

The authors declare that the research was conducted in the absence of any commercial or financial relationships that could be construed as a potential conflict of interest.

## Publisher’s Note

All claims expressed in this article are solely those of the authors and do not necessarily represent those of their affiliated organizations, or those of the publisher, the editors and the reviewers. Any product that may be evaluated in this article, or claim that may be made by its manufacturer, is not guaranteed or endorsed by the publisher.

## Supplementary Material

Supplementary Figure 1 Cycles included in the study.

Supplementary Figure 2 ROC curve analysis of HCG_12_ in predicting a live birth. Red line, biopsy group; green line, control group. AUCs are 0.886 (95% CI, 0.870–0.902) and 0.874 (95% CI, 0.864–0.885) for the biopsy group and control group, respectively (P<0.001). The points indicated by arrows represent the optimal cutoff values with the highest sum of sensitivity and specificity.
