# Supplementary figures and images for: Trophectoderm Biopsy Differentially Influences the Level of Serum β-Human Chorionic Gonadotropin With Different Embryonic Trophectoderm Scores in Early Pregnancy From 7847 Single-Blastocyst Transfer Cycles
Source: Front Endocrinol (Lausanne). 2022 Feb 18;13:794720. doi: 10.3389/fendo.2022.794720 (PMC8894721; doi:10.3389/fendo.2022.794720)

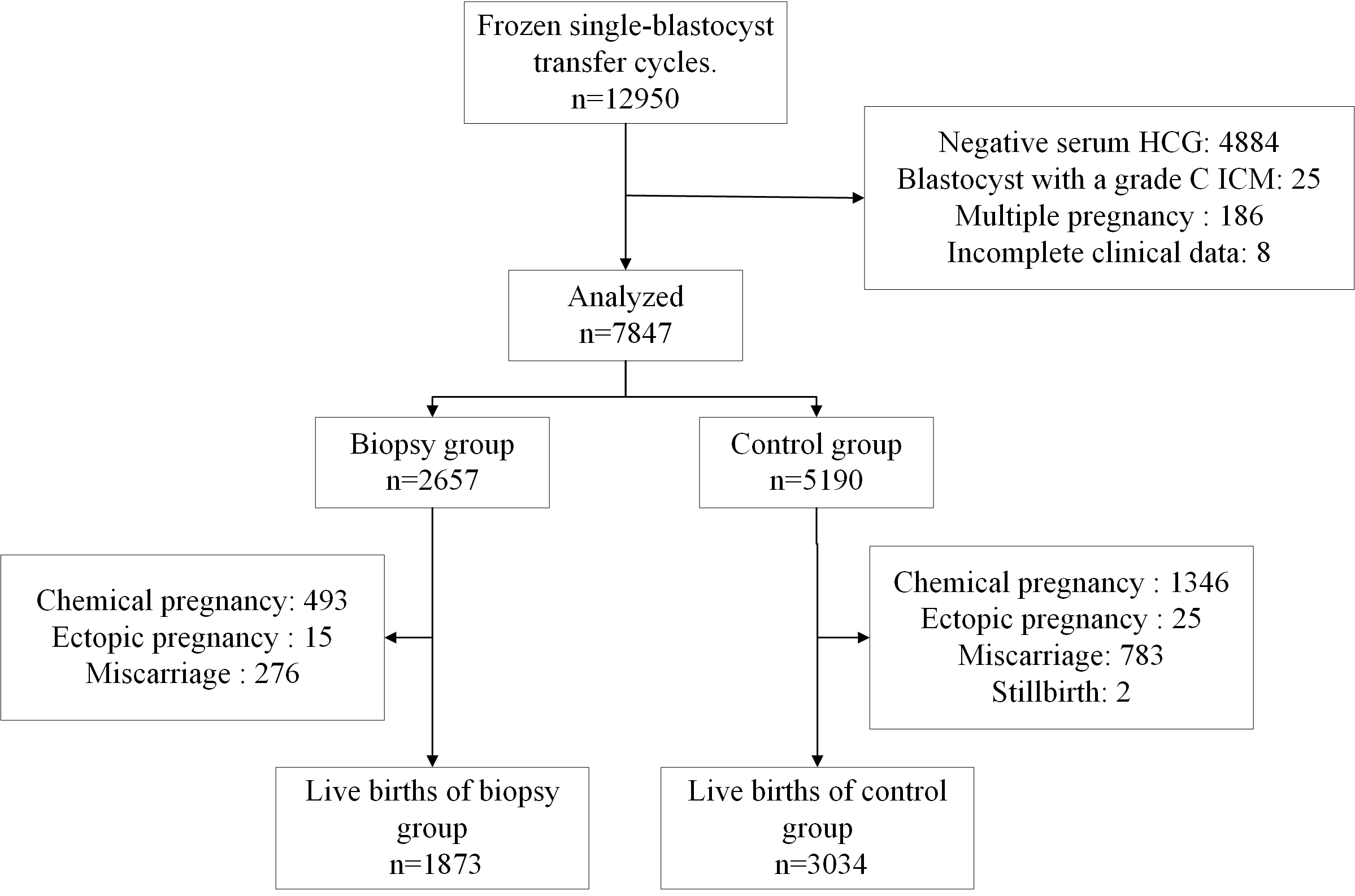

Supplement: Supplementary Figure 1 — Cycles included in the study. [file Image_1.jpeg]

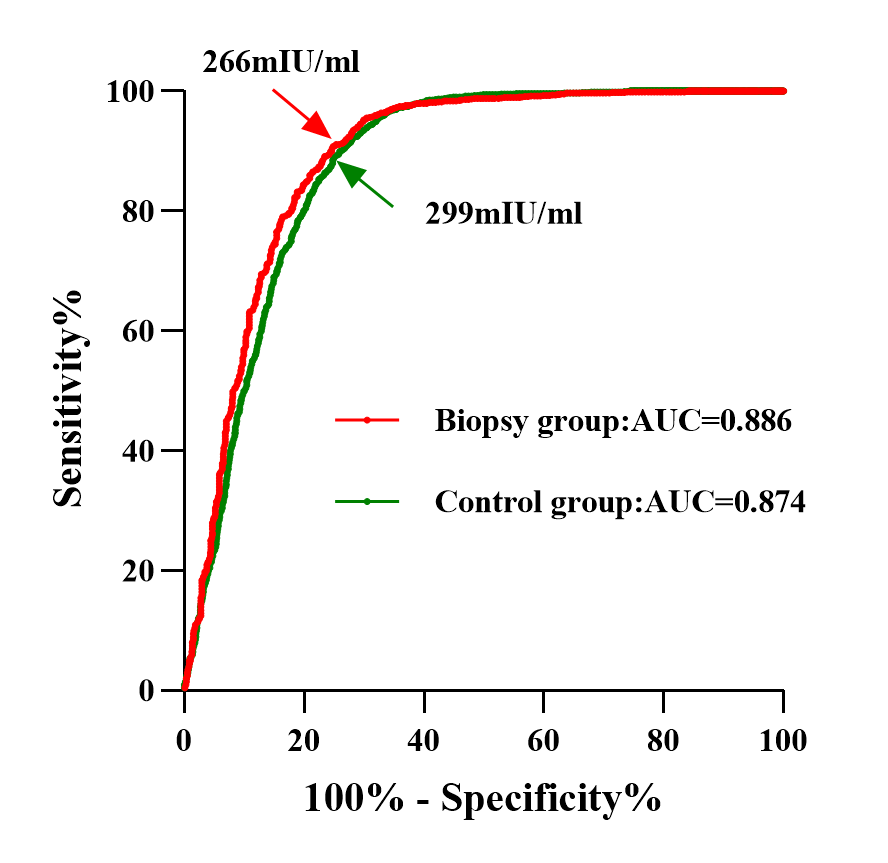

Supplement: Supplementary Figure 2 — ROC curve analysis of HCG12 in predicting a live birth. Red line, biopsy group; green line, control group. AUCs are 0.886 (95% CI, 0.870–0.902) and 0.874 (95% CI, 0.864–0.885) for the biopsy group and control group, respectively (P<0.001). The points indicated by arrows represent the optimal cutoff values with the highest sum of sensitivity and specificity. [file Image_2.tif]
