# Supplementary material for: Trophectoderm Biopsy Differentially Influences the Level of Serum β-Human Chorionic Gonadotropin With Different Embryonic Trophectoderm Scores in Early Pregnancy From 7847 Single-Blastocyst Transfer Cycles
Source: Front Endocrinol (Lausanne). 2022 Feb 18;13:794720. doi: 10.3389/fendo.2022.794720 (PMC8894721; doi:10.3389/fendo.2022.794720)
Supplement: Supplementary file 3 [file Table_1.docx]

**Supplementary Table 1** Baseline characteristics of different morphological TE-score subgroups relative to live births.

| TE morphological score | A | | |  | B | | |  | C | | |
| --- | --- | --- | --- | --- | --- | --- | --- | --- | --- | --- | --- |
| Group | Biopsy group | Control group | p.value |  | Biopsy group | Control group | p.value |  | Biopsy group | Control group | p.value |
| FET cycles, n | 231 | 216 |  |  | 1337 | 1824 |  |  | 305 | 994 |  |
| Age (years) | 33(29-36) | 31(28-34) | 0.002 |  | 33(30-37) | 31(29-34) | ＜0.001 |  | 33(29-37) | 31(29-34) | ＜0.001 |
| BMI (kg/m^2^) | 21.85(20.24-23.12) | 22.03(20.48-23.53) | 0.324 |  | 21.64(20.03-23.34) | 21.78(20.03-23.42) | 0.583 |  | 21.7(20.19-23.36) | 21.76(20-23.44) | 0.861 |
| Duration of infertility (years) | 2(1-4) | 3(2-5) | ＜0.001 |  | 2(1-4) | 3(2-5) | ＜0.001 |  | 2(1-4) | 3(2-5) | ＜0.001 |
| Basal values |  |  |  |  |  |  |  |  |  |  |  |
| FSH (mIU/mL) | 5.55(4.76-6.62) | 5.57(4.71-6.49) | 0.581 |  | 5.68(4.74-6.77) | 5.38(4.45-6.37) | ＜0.001 |  | 5.71(4.63-6.77) | 5.47(4.51-6.54) | 0.057 |
| LH (mIU/mL) | 3.74(2.46-4.98) | 3.51(2.49-4.91) | 0.610 |  | 3.52(2.57-4.68) | 3.41(2.36-4.85) | 0.111 |  | 3.29(2.34-4.46) | 3.26(2.24-4.68) | 0.930 |
| E_2_ (pg/mL) | 34.49(26-45.22) | 33(24-41.98) | 0.073 |  | 34(26-44.33) | 32(24-44) | 0.003 |  | 33(26.41-44) | 32(24-43) | 0.071 |
| Endometrial thickness on the day before transfer (mm) | 11.9(10.6-13.2) | 11.8(10.6-13) | 0.687 |  | 11.9(10.6-13.3) | 12(10.7-13.2) | 0.661 |  | 12(10.8-13.2) | 11.9(10.6-13.1) | 0.269 |
| Advanced age, %（n） | 36.36(84) | 18.98(41) | ＜0.001 |  | 39.87(533) | 20.12(367) | ＜0.001 |  | 41.31(126) | 23.54(234) | ＜0.001 |
| Chromosomal abnormality, %（n） | 31.6(73) | 0.93(2) | ＜0.001 |  | 29.77(398) | 0.99(18) | ＜0.001 |  | 35.08(107) | 1.01(10) | ＜0.001 |
| Monogenic disease, %（n） | 12.99(30) | 4.17(9) | 0.001 |  | 15.86(212) | 4.11(75) | ＜0.001 |  | 16.39(50) | 3.42(34) | ＜0.001 |
| RSA, %（n） | 27.71(64) | 1.39(3) | ＜0.001 |  | 28.87(386) | 3.02(55) | ＜0.001 |  | 22.3(68) | 2.11(21) | ＜0.001 |
| RIF, %（n） | 4.76(11) | 4.17(9) | 0.761 |  | 4.04(54) | 1.97(36) | 0.001 |  | 3.61(11) | 2.41(24) | 0.261 |
| Endometrial preparation protocols, %（n） |  |  | 0.001 |  |  |  | ＜0.001 |  |  |  | ＜0.001 |
| NC-FET | 88.74(205) | 75.46(163) |  |  | 87.43(1169) | 78.45(1431) |  |  | 88.52(270) | 78.37(779) |  |
| HT-FET | 7.79(18) | 17.13(37) |  |  | 6.96(93) | 12.23(223) |  |  | 4.92(15) | 10.36(103) |  |
| Down-regulating HT-FET | 3.46(8) | 7.41(16) |  |  | 5.61(75) | 9.32(170) |  |  | 6.56(20) | 11.27(112) |  |

Note: Variables are expressed as medians (interquartile range) unless otherwise stated. FET = frozen embryo transfer; BMI = body mass index; FSH = follicle-stimulating hormone; LH = luteinizing hormone; E_2_ = estradiol; RSA = recurrent spontaneous abortion; RIF = recurrent implantation failure； NC = natural cycle; HT = hormone therapy;
