# Supplementary material for: Trophectoderm Biopsy Differentially Influences the Level of Serum β-Human Chorionic Gonadotropin With Different Embryonic Trophectoderm Scores in Early Pregnancy From 7847 Single-Blastocyst Transfer Cycles
Source: Front Endocrinol (Lausanne). 2022 Feb 18;13:794720. doi: 10.3389/fendo.2022.794720 (PMC8894721; doi:10.3389/fendo.2022.794720)
Supplement: Supplementary file 4 [file Table_2.docx]

**Supplementary Table 2** ROC curve analysis of HCG_12_ in predicting a live birth with different grades of TE-scored blastocyst transfers.

| Group | TE morphological scores | AUC (95%CI) | Cutoff value | Sensitivity | Specificity | p-value |
| --- | --- | --- | --- | --- | --- | --- |
| Biopsy group | A | 0.863(0.802~0.924) | 366 | 87.9% | 77.6% | <0.001 |
|  | B | 0.873(0.851~0.895) | 228 | 94.5% | 69.6% | <0.001 |
|  | C | 0.911(0.885~0.937) | 192 | 92.5% | 78.7% | <0.001 |
| Control group | A | 0.863(0.814~0.912) | 343 | 92.1% | 70.7% | <0.001 |
|  | B | 0.853(0.836~0.869) | 299 | 90.8% | 70.4% | <0.001 |
|  | C | 0.893(0.879~0.908) | 203 | 94.3% | 72.7% | <0.001 |

Note. AUC = area under the curve; CI = confidence interval.
